# Subjective cognitive decline is associated with a higher risk of objective cognitive decline: A cross-sectional and longitudinal study

**DOI:** 10.3389/fpsyt.2022.950270

**Published:** 2022-09-29

**Authors:** Wei Li, Ling Yue, Shifu Xiao

**Affiliations:** ^1^Department of Geriatric Psychiatry, Shanghai Mental Health Center, Shanghai Jiao Tong University School of Medicine, Shanghai, China; ^2^Alzheimer's Disease and Related Disorders Center, Shanghai Jiao Tong University, Shanghai, China

**Keywords:** SCD, RMFG, cognition, MRI, cohorts

## Abstract

**Background:**

Subjective cognitive decline (SCD) is considered as an independent risk factor for objective cognitive impairment, such as dementia and mild cognitive impairment (MCI), but the mechanism is unclear.

**Methods:**

The current study consisted of two parts, the first of which included 1,010 older adults with SCD and 535 normal controls and was followed for 1 year. The second cross-sectional study included 94 older adults with SCD and 64 healthy controls. Unlike the first cohort, subjects in the second study underwent magnetic resonance imaging and had more detailed neuropsychological tests, such as Mini- mental State Examination (MMSE), Montreal Cognitive Assessment (MoCA), Digit Span, Auditory Verbal Learning Test (AVLT), Associative Learning Test (ALT), Verbal Fluency (VF), Wechsler's filling and Wechsler's building blocks.

**Results:**

In cohort 1, we found that SCD had a higher risk of objective cognitive impairment compared to normal controls (X^2^ = 20.354, *p* = 0.002), and the results of Cox Regression analysis also suggest that SCD was a risk factor for objective cognitive decline (*p* < 0.001, HR = 2.608, 95%CI: 2.213–3.075). In study 2, we found that the scores of MoCA, digit span, verbal fluency, and Wechsler's filling of SCD elderly were significantly lower than those of normal controls, but the cortical thickness of the rostral middle frontal gyrus (RMFG) was significantly higher than that of normal controls (*p* < 0.05).

**Conclusions:**

SCD is a cognition-related disease with multi-cognitive domain impairment, which is associated with a higher risk of objective cognitive impairment. Moreover, the increased cortical thickness of the left rostral middle frontal gyrus (RMFG) might be an important mechanism of cognitive decline in SCD.

## Introduction

Subjective cognitive decline (SCD) refers to individuals' perceived decline in memory and/or other cognitive function relative to their previous level of performance, without objective neuropsychological deficits (overall cognitive function) (1). The accumulated evidence shows that SCD, manifests prior to the onset of clinical impairment, and has an increased risk for future mild cognitive impairment (MCI) and dementia (2). Individuals with SCD often present brain abnormalities reminiscent of Alzheimer disease (AD), including disrupted functional connectivity, increased cerebral β-amyloid [Aβ] deposition, as well as greater atrophy and glucose hypometabolism in AD signature regions (2, 3). Since assessment of SCD is less invasive and expensive than measure cerebrospinal fluid or neuroimaging biomarkers, SCD has the potential to become a biomarker of AD (4).

The prevalence of SCD varies greatly (from 12.3 to 84.5%) due to the differences in definitions (pre-MCI SCD or SCD in all stages of the disease), assessment methods (using single question of perceived memory problem or evaluation using several questions) and the study population (community-dwelling or clinical population) (5–8). However, not all individuals with subjective cognitive decline will necessarily turn into objective cognitive impairment, such as dementia or mild cognitive impairment (MCI) (9). Therefore, early identification of SCD patients who are likely to progress to objective cognitive decline and make a tailored diagnostic procedure is crucial, this is because early diagnosis and early intervention can effectively improve the prognosis of SCD patients.

Neuropsychological test and magnetic resonance imaging (MRI) are both powerful tools to study cognitive function. By combining the two, it is expected to understand the cognitive characteristics of individuals with SCD and the possible biological mechanisms. In our previous study, we found that the asymmetries in the left and right hippocampus and amygdala might be developed as biomarkers for SCD (10). Moreover, Thomas KR et al. found that hyper perfusion in the rostral middle frontal gyrus (RMFG) is also an important clinical feature of SCD (11). Therefore, the hippocampus, amygdala and RMFG may be developed as biomarkers of SCD. In the current study, we will use two cohorts to study SCD: one is to compare the risk of future objective cognitive impairment between individuals with SCD and normal controls; the other is dedicated to exploring the cognitive and imaging characteristics in individuals with SCD. Our study hypothesized that (1) SCD has a higher risk of future cognitive decline than normal controls. (2) Although the overall cognitive function of SCD is no different from that of the normal elderly, they may have significant differences in the volumes of certain brain regions such as hippocampus, amygdala and RMFG.

## Materials and methods

### Participants

The current study consisted of two studies, one from the China Longitudinal Aging Study (CLAS) (12) and the other from Shanghai brain health foundation (SHBHF2016001) (13). In cohort 1, 20 target communities (i.e, 18 urban and 2 rural) located in the eastern, western, and mid parts of China have been included in this study. According to the 2010 National Census, permanent residents aged 60 and over were entered into the database. A simple random sample comprising 4,411 residents was selected to identify potential participants, while 3,514 participants completed the baseline survey. Next, 1,010 participants with subjective cognitive decline (SCD) and 535 older adults without cognitive impairment were included in our final study. All the participants underwent a baseline screening process that included physical and neurological examinations, medical history, and cognitive assessments. Moreover, they also completed a 1-year follow-up study with the same procedures as baseline.

The second study included 94 SCD individuals and 64 normal controls from the Shanghai brain health foundation (SHBHF2016001). The project was launched in 2016 as a prospective and observational cohort study. The specific content of this project includes understanding the mortality, prevalence, morbidity and population distribution characteristics of mild cognitive impairment and Alzheimer's disease in the elderly over 60 years of age in Shanghai community. Difference from the first cohort, they all underwent structural MRI of the T1 phase but without follow up. Participants in both the first and second studies must meet the following requirements: (1) Han Chinese. (2) ≥60 years old. (3) absence of mild cognitive impairment (MCI) or dementia. (4) without serious physical or mental illness. (5) able to complete the study. Those, who were (1) < 60 years old. (2) suffered from severe visual or hearing impairment; and (3) refusing to cooperate with the investigation were excluded.

Ethical approval was issued by Shanghai Mental Health Center, and all the participants had signed an informed consent before the study was initiated.

### Clinical assessment and diagnostic criteria

#### Subjective cognitive decline

The diagnosis of subjective cognitive decline (SCD) was based on a conceptual framework of criteria for identification of SCD (14): (1) self -reported cognitive decline (Information was obtained through a standardized questionnaire, which asked: 1. Do you think you have memory loss? 2. If so, for years). 2 the onset age was more than 60 years old. (2) the presence of gradual memory decline had persisted for ≥6 months; (3) objective cognitive score in normal range [the optimal cutoff scores of the MoCA for the groups aged ≤75 years old and education ≤6 years, aged >75 years old and education ≤6 years, aged ≤75years old and education >6years, aged >75 years old and education >6 years in screening for MCI were identified as 19.5, 15.5, 24.5 and 24.5, respectively, and the optimal cutoff scores for dementia were 18.5, 10.5, 18.5 and 20.5, respectively (15)].

#### Cognitively unimpaired

Participants were considered cognitively unimpaired if there were: (a) without subjective memory or other cognitive discomfort; (b) without evidence of a history of memory or other cognitive decline; (c) The overall cognitive score is in normal range (MoCA≥25) (16).

#### Mild cognitive impairment and dementia

The diagnosis of MCI was based on the diagnosis standard of Petersen (17), while the diagnosis of dementia was based on the Diagnostic and Statistical Manual of mental disorders, Fourth Edition (DSM-IV) (18).

The Clinical diagnosis of all subjects will be performed by two experienced attending geriatrics physicians, and if the two patients' diagnosis is inconsistent, the diagnosis will be reviewed by the chief physician.

### Neuropsychological tests

The Mini- mental State Examination (MMSE) (19) and Montreal Cognitive Assessment (MoCA) (20) were used to assess the subjects' overall cognitive function, while Digit Span (21), Auditory Verbal Learning Test (AVLT) (22), Associative Learning Test (ALT) (23), Verbal Fluency (VF) (24), Wechsler's filling and Wechsler's building blocks (25) were used to assess their working memory storage (digit span) (26), episodic verbal memory (AVLT) (27), associative learning (ALT) (28), language, memory and executive functioning (VF) (29), and executive functioning (Wechsler's filling and Wechsler's building blocks), respectively. In cohort 1, the scales used included MMSE and MoCA, while study 2 included all of the neuropsychological tests mentioned above. A brief introduction to the above scales and detailed procedures can be found in our previously published article (12, 25). All operations were performed by conformance trained evaluators, and the entire process would be recorded for subsequent evaluation of the operation quality.

### MR image acquisition and processing

T1-Brain structure image was acquired by using a Siemens Magnetom Verio 3.0T scanner (Siemens, Munich, Germany). The parameters of T1-weighted 3D magnetization prepared rapid gradient echo (MPRAGE) sequences were as follows: TR = 2,300 ms, TE = 2.98 ms, matrix size = 240 × 256; flip angle of 9 degree, field of view (FOV) = 240 × 256 mm; slice thickness = 1.2 mm. Volumetric data was assessed by automated procedures, which have been described by Wolz R et al. (30). For each subject, volume and asymmetry with various brain areas as well as cortical thickness were extracted directly using FreeSurfer v6.0. Based on our previous research basis and previous literature (10, 31, 32), we took the volume of hippocampus, amygdala and the cortical thickness of rostral middle frontal gyrus as our target research objects.

### Covariates

General demographic information was gathered by self-reported, and the following data, such as age, gender, education, smoking, drinking, tea drinking, taking exercise, hobby, diabetes and hypertension were collected by standardized questionnaire. Those variables that differed between SCD and normal controls were considered as covariates (In addition to gender, age, education and other variables that are recognized to have an impact on cognitive function, tea consumption was also considered as a covariate, as there was a certain difference in tea consumption between the SCD group and the normal control group).

### Follow-up (Incident objective cognitive impairment)

All individuals included in the final study (*n* = 1545) were evaluated at baseline and followed up for 1 year. In the SCD group (*n* = 1010), 90 progressed into amnesic mild cognitive impairment (aMCI), 11 into vascular mild cognitive impairment (vMCI), 8 into Aizheimer's disease (AD), 6 into vascular dementia (VD), 2 into mixed dementia (MD). In normal controls group (*n* = 535), 17 progressed into aMCI, 4 into vMCI, 2 into AD, 4 into VD, 4 into MD. Then we assigned all cases of cognitive decline (including aMCI, vMCI, SCI, AD, VD, and MD) into the cognitive decline group (whether the subjects were vascular or non-vascular was mainly determined by the score of the ischemia index scale and whether the patients had risk factors for vascular diseases, such as hypertension and stroke, in addition, the subtypes of dementia and MCI are diagnosed based on clinical and neuropsychological tests, such as MOCA, AVLT, VF and so on).

### Statistical analysis

Continuous variables were expressed as mean ± standard deviation (SD), and categorical variables were expressed as frequencies (%). A single sample Kolmogorov-Smirnov test was used to test whether data conforms to normal distribution. Next, Independent sample *t*-test and Mann–Whitney tests were respectively used to compare the normal data and non-normal data between the SCD group and the normal control group, while Chi-square tests was used to compare those classification variables. Then multiple stepwise Cox regression analysis (Model 1 contains only SCD; Model 2 contains SCD, age, gender and education; Model 3 contains SCD, age, gender, education, and tea drinker) was used to further explore the relationship between SCD and further objective cognitive decline (controlled for other relevant variables) (Cohort 1). Moreover, a linear regression analysis (mediating model) was also performed to investigate the association among SCD, cognitive-related brain areas, and cognitive scores in study 2 [The first step was to investigate the regression analysis of independent variable (SCD) and intermediate variable (the cortical thickness of the left rostral middle frontal gyrus), if the *p*-value was < 0.05, enter the next step; The second step was to do the regression of SCD to MoCA scores, and the regression of SCD and the cortical thickness of the left rostral middle frontal gyrus to MoCA scores by two models; The third step, if the *p*-value of the regression model of SCD to MoCA scores was > 0.05, then the significance test was not passed, and if the *p*-value of the regression model was < 0.05, then, the regression model coefficient between the cortical thickness of the left rostral middle frontal gyrus and MoCA scores was examined. If its *P*-value was still < 0.05, it was considered that the cortical thickness of the left rostral middle frontal gyrus played a mediating effect between SCD and MoCA scores]. All mediation analysis models had been corrected by Bonferroni's analysis. All the statistical analyses were performed using SPSS 22.0 (IBM Corporation, Armonk, NY, United States), and two-tailed tests were performed at a significance level of *P* < 0.05.

## Results

### Characteristic of subjects with different cognitive states (cohort 1)

Compared with normal controls, individuals with subjective cognitive decline had fewer years of schooling and a lower proportion of tea consumption (*p* < 0.05), while there were no statistical differences (*p* > 0.05) in age, gender, smoker, drinker, take exercise, hobby, hypertension, diabetes, baseline MMSE and baseline MoCA. Table 1 presents the results.

**Table 1:** Comparison of baseline general demographic data between subjective cognitive impairment and normal controls.

**Characteristics**	**SCD** **(*n* = 1010)**	**Normal** **(*n* = 535)**	**X^2^ OR T**	***P***
Age, y	70.47 ± 7.45	70.08 ± 7.77	0.895	0.371
Education, y	9.72 ± 5.07	8.92 ± 5.02	2.903	0.004*
Male, *n* (%)	483(47.8)	269(50.3)	0.846	0.364
Smoker, *n* (%)	282(27.9)	161(30.1)	0.807	0.376
Drinker, *n* (%)	199(19.7)	110(20.6)	0.161	0.689
Tea drinker, *n* (%)	495(49.0)	298(55.7)	6.268	0.014*
Take exercise, *n* (%)	758(75.0)	414(77.4)	1.040	0.318
Hobby, *n* (%)	640(63.4)	360(67.3)	2.358	0.131
Hypertension, *n* (%)	484(47.9)	245(45.8)	0.635	0.453
Diabetes, *n* (%)	145(14.4)	83(15.5)	0.373	0.547
Baseline MMSE	27.12 ± 3.21	26.83 ± 3.48	1.641	0.101
Baseline MoCA	23.17 ± 4.85	22.87 ± 5.32	1.090	0.276

* means p < 0.05; MMSE means Mini-mental State Examination.

MoCA means Montreal Congnitive Assessment.

### The results of the multiple cox regression model (cohort 1)

**Figure 1:**
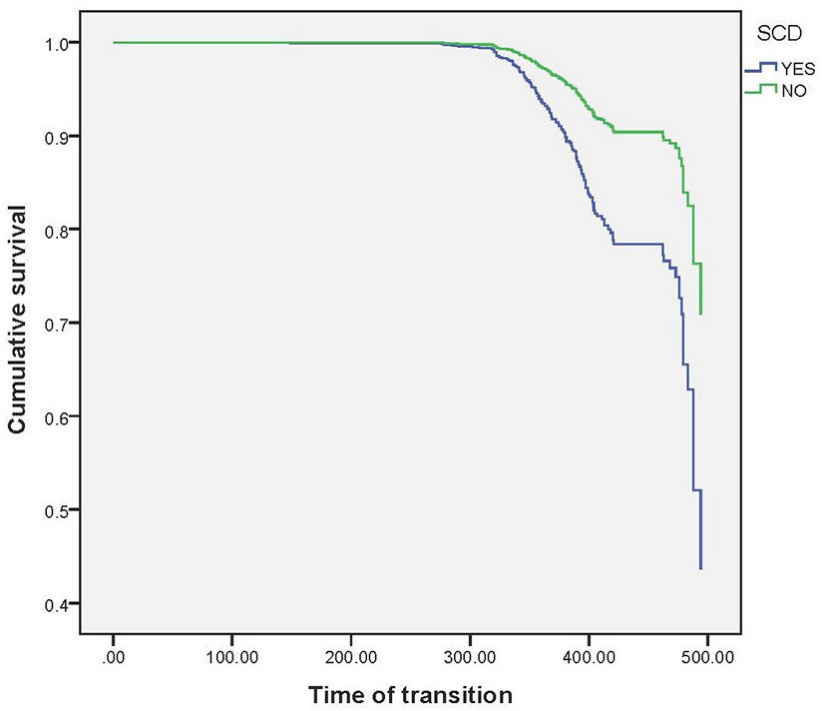
Baseline subjective cognitive decline as a survival function of future cognitive decline.

By chi-square test, we found that SCD had a higher risk of objective cognitive impairment compared to normal controls (X^2^ = 20.354, *p* = 0.002) (The normal control group was from the same cohort as SCD to ensure no time error and sampling error). Then Multiple Cox regression model was used to explore the relationship between SCD and future cognitive decline (Objective cognitive decline was regarded as the dependent variable, and transition time was taken as the time variable). Model 1 did not control any variables, and the results showed that SCD was a risk factor for objective cognitive decline (*p* < 0.001, HR = 2.620, 95%CI: 2.255–3.044); Model 2 controlled some variables, such as age, gender, education, and Model 3 furtherly controlled other variables, such as tea drinker, and different statistical models still did not change the statistical results (Table 2). The results of the survival curve suggested that older adults with baseline SCD would develop objective cognitive impairment earlier and more often. Figure 1 presents the results.

**Table 2:** Association between baseline subjective cognitive disorder and future cognitive change (results of COX regression analysis).

**Variables**	**B**	**S.E**	**Wald**	**df**	**p**	**HR**	**95% confidence interval**	
Model 1								
SCD	0.963	0.077	158.273	1	< 0.001*	2.620	2.255	3.044
Model 2								
SCD	0.988	0.084	140.059	1	< 0.001*	2.686	2.281	3.164
Education	0.011	0.007	2.464	1	0.116	1.011	0.997	1.025
Age	0.016	0.004	15.291	1	< 0.001*	1.016	1.008	1.024
Male	−0.167	0.065	6.658	1	0.010*	0.847	0.746	0.961
Model 3								
SCD	0.959	0.084	130.443	1	< 0.001*	2.608	2.213	3.075
Education	0.010	0.007	2.216	1	0.137	1.010	0.997	1.024
Tea drinker	−0.229	0.066	12.202	1	< 0.001*	0.795	0.699	0.904
Age	0.014	0.004	12.491	1	< 0.001*	1.014	1.006	1.022
Male	−0.094	0.068	1.903	1	0.168	0.911	0.797	1.040

Model 1 contains only SCD;

Model 2 contains SCD, age, gender, and education.

Model 3 contains SCD, age, gender, education, and tea drinker.

SCD means Subjective cognitive disorder;

* mean p < 0.05.

### Results associated with neuropsychological tests and structural magnetic resonance (study 2)

To explore the possible mechanism of SCD affecting cognitive function, in this part, we randomly selected 158 people (SCD, *n* = 94; normal controls, *n* = 64), all of whom completed MRI. The random method is to use the SPSS's random number generator. The SCD group and the normal control group were matched in age, education, males, smokers, drinkers, tea drinker, take exercise, hobbies, hypertension, and diabetes (*p* > 0.05).

**Figure 2:**
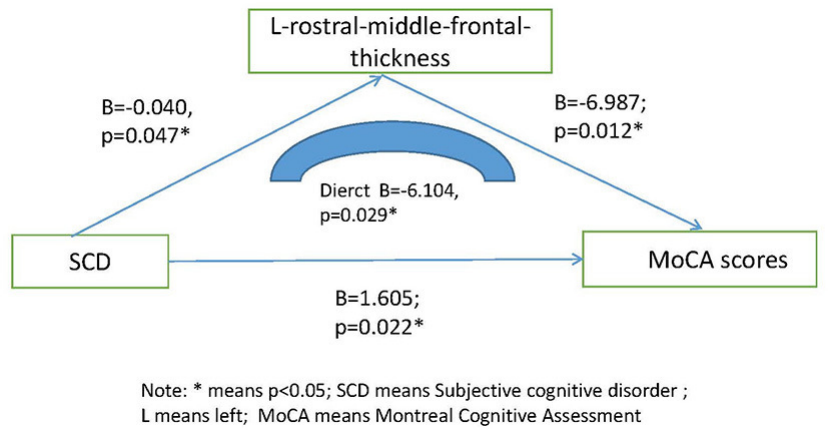
Mediating effect model among SCD, L-rostral-middle-frontal-thickness and MoCA scores. * means *p* < 0.05; SCD means subjective cognitive disorder; L means left; moca means Montreal Cognitive Disorder.

The scores of SCD individuals on MoCA, digit span, verbal fluency, and Wechsler's filling were significantly lower than those of normal controls (*p* < 0.05), but there was no statistical difference (*p* > 0.05) between the two groups on MMSE, auditory verbal learning test, associative learning test and Wechsler's building blocks. However, we found that the cortical thickness of the rostral middle frontal gyrus (RMFG) in SCD individuals was significantly higher than that of normal controls (*p* < 0.05), while there was no statistical difference (*p* > 0.05) in total brain volume, left hippocampus, right hippocampus, left amygdala and right amygdala between the two groups. Table 3 presents the results. Through linear regression analysis (the mediation model), we found that the cortical thickness of the left rostral middle frontal gyrus (RMFG) may affect cognitive function (B = −6.104, *p* = 0.029) and therefore self-reports of decline (SCD). Figure 2 presents the results. In the statistical process, we have controlled for the effects of gender, age and education on the results of the study.

**Table 3:** Comparison of general demographic data, neuropsychological tests, and structural MRI between SCD and normal controls.

**Variables**	**SCD** **(*n* = 94)**	**Normal** **(*n* = 64)**	**X^2^or t**	**p**
Age,y	67.07 ± 5.567	67.59 ± 6.423	−0.541	0.590
Education, y	8.67 ± 3.425	8.95 ± 3.873	−0.470	0.639
Male, *n* (%)	37(39.4)	35(54.7)	3.606	0.074
Smoker, *n* (%)	31(33.0)	17(26.6)	0.741	0.481
Drinker, *n* (%)	19(20.2)	12(18.8)	0.052	1.000
Tea drinker, *n* (%)	32(34.0)	26(40.6)	0.710	0.407
Take exercise, *n* (%)	57(60.6)	46(71.9)	2.119	0.175
Hobby, *n* (%)	58(61.7)	42(65.6)	0.252	0.737
Hypertension, *n* (%)	47(50.0)	26(40.6)	1.346	0.260
Diabetes, *n* (%)	18(19.1)	8(12.5)	1.224	0.382
Neuropsychological tests				
MMSE	27.70 ± 2.204	28.14 ± 1.740	−1.333	0.185
MoCA	23.82 ± 4.632	25.42 ± 3.685	−2.312	0.022*
Digit Span	14.26 ± 3.997	16.33 ± 4.190	−3.138	0.002*
Auditory verbal learning test	31.94 ± 8.040	34.66 ± 10.365	−1.768	0.080
Associative learning test	6.51 ± 3.098	6.80 ± 3.282	−0.561	0.576
Verbal fluency	27.08 ± 7.362	30.33 ± 10.872	−2.087	0.039*
Wechsler's filling	10.20 ± 3.552	11.92 ± 4.373	−2.614	0.010*
Wechsler's building blocks	27.14 ± 7.861	29.20 ± 6.646	−1.710	0.089
Structural magnetic resonance imaging				
Total brain volume,cm^3^	1433.35 ± 155.33	1464.12 ± 141.50	−1.267	0.207
Left hippocampus, cm^3^	3.601 ± 0.454	3.700 ± 0.411	−1.392	0.166
Right hippocampus, cm^3^	3.833 ± 0.496	3.908 ± 0.437	−0.979	0.329
Left amygdala, cm^3^	1.528 ± 0.234	1.547 ± 0.222	−0.508	0.612
Right amygdala, cm^3^	1.663 ± 0.256	1.718 ± 0.246	−1.343	0.181
L–rostral–middle–frontal–thickness, mm^3^	2.29 ± 0.131	2.25 ± 0.109	2.002	0.047*
R–rostral–middle–frontal–thickness, mm^3^	2.28 ± 0.139	2.23 ± 0.113	2.679	0.008*

* means p < 0.05; SCD means Subjective cognitive disorder; MMSE means mini–mental state examination.

MoCA means Montreal Cognitive Assessment; L means left, R means right.

## Discussion

In this study, we used two cohorts to explore the cognitive characteristics of subjective cognitive decline (SCD) and its risk of developing objective cognitive impairment in the future, and to explore the radiological mechanisms by which SCD increases cognitive decline.

Finally, we found that: (1) compared with the normal control, SCD had a loss in multiple cognitive domains and a higher risk of objective cognitive decline. (2) increased cortical thickness of the left rostral middle frontal lobe might lead to decreased cognitive function and ultimately might contribute to the development of SCD.

Older adults with subjective cognitive decline (SCD) are increasingly considered to be at risk for non-normative cognitive decline (33). However, similar large-scale longitudinal follow-up studies have been relatively rare in China, for example, in Qi et al.' study, they found that both self- and informant-reported memory complaints were associated with an increased risk of cognitive decline and cognitive impairment conversion, especially in persons with male gender and high educational background (34). In Yue et al.' study, they found that a history of stroke, a low education level, a low baseline MoCA score, a shrunk left amygdala, and enlarged white matter at the banks of the right superior temporal sulcus were found to would promote the transition from SCD to MCI (35). In our study, we also found that older adults with subjective cognitive decline at baseline were at higher risk for objective cognitive impairment in the future. However, due to the heterogeneity of SCD, not all patients with SCD necessarily progress to Alzheimer's disease. For example, in Feifei Jia et al.' study, they followed 2,099 cognitively normal adults aged 65 or over for 2 years and found that baseline subjective cognitive decline significantly increased the risk of dementia. While in Schwilk et al.'s study, they followed 28 older adults for 10 years and found that none of them developed mild cognitive impairment or dementia (36). Therefore, it is necessary to further study the relationship between subjective cognitive impairment and MCI or dementia, and the addition of Alzheimer's disease-related biomarkers to SCD may be more helpful in revealing the association.

According to the diagnostic framework, the objective neuropsychological assessment of SCD was within the normal range. However, SCD still showed a higher transition risk of MCI or dementia (2). Therefore, effective differentiation between SCD and normal elderly people is of clinical importance because it will help to give patients early intervention and improve their prognosis. Neuropsychological testing is an effective tool for neuroscience research and helps to reveal the cognitive characteristics of different diseases, such as dementia and Parkinson's disease (37, 38). In our current study, we used a series of neuropsychological scales to assess subjects' cognitive characteristics, including MMSE, MoCA, digit span, auditory verbal learning test (AVLT), associative learning test (ALT), verbal fluency (VF), Wechsler adult Intelligence scale (WAIS)-III Block Design and Wechsler adult Intelligence scale, and finally found that SCD individuals had lower scores on MoCA total score, digit span, verbal fluency, and Wechsler adult Intelligence scale than the normal population. These results suggest that SCD patients have significant impairment of overall cognitive function, immediate memory, verbal fluency memory and executive function. In Liew TM et al.' study, they found that there was a significant difference in cognitive performance between SCD patients with memory complaints and those with non-memory complaints, and their prognosis was also different (39). In Hao et al.'s study, they also found that the scores of auditory verbal learning test-long delayed recall and MoCA-B were lower in the SCD group than those in the normal control group (40, 41). Therefore, SCD, although not a disease, carries a higher risk of progressing into MCI or dementia.

To explore the possible mechanisms by which SCD affects cognitive function, we added structural magnetic resonance in the second cohort. We found that the cortical thickness of the rostral middle frontal gyrus (RMFG) in SCD individuals was significantly higher than that of normal controls, However, we did not find differences in hippocampal volume and amygdala volume between SCD patients and normal elderly people, indicating that the two couldn't be used as biomarkers to distinguish SCD from normal elderly people. Through linear regression analysis and mediation model, we found that the cortical thickness of the left rostral middle frontal might mediate worse performance on MoCA and other tests (such as learning or verbal fluency tasks) in patients with SCD. Cortical thickness of the rostral middle frontal gyrus (RMFG), a region critical for executive function, including attention, planning, working memory, executive cognition, and emotion regulation, has been associated with stress and depression -related phenotypes (32). In Youn et al.'s study, they found that the cortical thickness of the rostral middle frontal was significantly reduced in individuals with SCD, and cortical thickness in this region was significantly correlated with cognitive score (42). In Nigro S's study, they also found that patients with behavioral variant frontotemporal dementia displayed lower values of local efficiency in the cortical thickness of rostral middle frontal gyrus (43). However, until now, there has been no research report on the correlation between RMFG and learning and language function. So we can't tell if our findings are consistent with those of others. We speculate that the association between SCD and thicker cortical thickness of the rostral middle frontal gyrus (RMFG) may also be related to the possibility that individuals with better cognition may be more likely to be aware of subtle cognitive decline and report SCD (39). Another possibility is that the current diagnostic system of SCD may have some problems, and simply relying on clinical diagnosis and neuropsychological tests may not reflect the nature of the whole disease. Therefore, in future studies, we will further explore the association between brain structure and cognitive function in SCD patients with different memory complaints.

## Conclusions

The elderly with subjective cognitive decline have multiple cognitive impairment and higher risk of objective cognitive decline, and the increased the cortical thickness of the rostral middle frontal gyrus (RMFG) is likely to be the core factor of the overall cognitive decline.

## Limitations

We have to admit that there are some limitations in our research. First, these data were from two totally different studies and therefore might not accurately represent the general older adult Chinese population. Second, we only followed the participants for 1 year, so the short follow-up was a major limitation of our study.

## Data availability statement

The raw data supporting the conclusions of this article will be made available by the authors, without undue reservation.

## Ethics statement

The studies involving human participants were reviewed and approved by Shanghai Mental Health Center. The patients/participants provided their written informed consent to participate in this study.

## Author contributions

WL and LY contributed to the study concept and design. SX analyzed the data and drafted the manuscript. All authors read and approve the final manuscript.

## Funding

This study was supported by grants from the clinical research center project of Shanghai Mental Health Center (CRC2017ZD02), Shenkang Clinical Innovation Project (TM201919), Clinical Research plan of SHDC (SHDC2020CR1038B), the Cultivation of Multidisciplinary Interdisciplinary Project in Shanghai Jiaotong University (YG2019QNA10), the Feixiang Program of Shanghai Mental Health Center (2018-FX-05 and 2020-FX-03), the National Natural Science Foundation of China (82001123 and 82101564), Shanghai Clinical Research Center for Mental Health (SCRC-MH and 19MC1911100), Shanghai Brain Health Foundation (SHBHF2016001) and the Shanghai Science and Technology Committee (20Y11906800). This project was also funded by the Shanghai brain health foundation (SHBHF2016001).

## Conflict of interest

The authors declare that the research was conducted in the absence of any commercial or financial relationships that could be construed as a potential conflict of interest.

## Publisher's note

All claims expressed in this article are solely those of the authors and do not necessarily represent those of their affiliated organizations, or those of the publisher, the editors and the reviewers. Any product that may be evaluated in this article, or claim that may be made by its manufacturer, is not guaranteed or endorsed by the publisher.
